# The Health History of First-Degree Relatives’ Dyslipidemia Can Affect Preferences and Intentions following the Return of Genomic Results for Monogenic Familial Hypercholesterolemia

**DOI:** 10.3390/genes15030384

**Published:** 2024-03-21

**Authors:** Tomoharu Tokutomi, Akiko Yoshida, Akimune Fukushima, Kayono Yamamoto, Yasushi Ishigaki, Hiroshi Kawame, Nobuo Fuse, Fuji Nagami, Yoichi Suzuki, Mika Sakurai-Yageta, Akira Uruno, Kichiya Suzuki, Kozo Tanno, Hideki Ohmomo, Atsushi Shimizu, Masayuki Yamamoto, Makoto Sasaki

**Affiliations:** 1 Iwate Tohoku Medical Megabank Organization, Iwate Medical University, Shiwa 020-3694, Japan; akikoyos@iwate-med.ac.jp (A.Y.);; 2 Department of Clinical Genetics, School of Medicine, Iwate Medical University, Morioka 020-8505, Japan; 3 Tohoku Medical Megabank Organization, Tohoku University, Sendai 980-8573, Japan

**Keywords:** familial hypercholesterolemia, genetic-testing preferences, family health history, information sharing, personalized healthcare

## Abstract

Genetic testing is key in modern healthcare, particularly for monogenic disorders such as familial hypercholesterolemia. This Tohoku Medical Megabank Project study explored the impact of first-degree relatives’ dyslipidemia history on individual responses to familial hypercholesterolemia genomic results. Involving 214 participants and using Japan’s 3.5KJPN genome reference panel, the study assessed preferences and intentions regarding familial hypercholesterolemia genetic testing results. The data revealed a significant inclination among participants with a family history of dyslipidemia to share their genetic test results, with more than 80% of participants intending to share positive results with their partners and children and 98.1% acknowledging the usefulness of positive results for personal health management. The study underscores the importance of family health history in genetic-testing perceptions, highlighting the need for family-centered approaches in genetic counseling and healthcare. Notable study limitations include the regional scope and reliance on questionnaire data. The study results emphasize the association between family health history and genetic-testing attitudes and decisions.

## 1. Introduction

Genetic testing, especially for monogenic disorders such as familial hypercholesterolemia (FH), is integral to modern healthcare as it provides deep insights into individual and family health risks. FH, characterized by elevated low-density lipoprotein cholesterol (LDL-C), markedly increases coronary artery disease risk; therefore, its early detection and management is vital for effective interventions and prevention [1].

Recent studies highlight the significance of familial genetic predispositions in disease occurrence [2,3,4]. Khera et al. [5] revealed the combined impact of genetic risks and lifestyle factors on coronary diseases, emphasizing the value of genetic testing in health-risk management. Furthermore, the influence of family health history on attitudes toward genetic testing and result sharing is gaining attention. Hunter et al. [6] reported common motives for sharing genetic test results, including informing relatives about genetic risks and personal interest in the findings.

In FH, genetic-testing results extend beyond the individual to the family, especially when there is a known history of related health issues. This study, aligning with the Tohoku Medical Megabank (TMM) Project [7], delves into how the dyslipidemia history of first-degree relatives impacts information-sharing preferences and intentions after receiving monogenic FH genetic results. This study contributes to understanding the role of familial health history in genetic-testing results and enriches our comprehension of familial data utilization.

## 2. Materials and Methods

### 2.1. Study Population and Participant Recruitment

**Figure 1:**
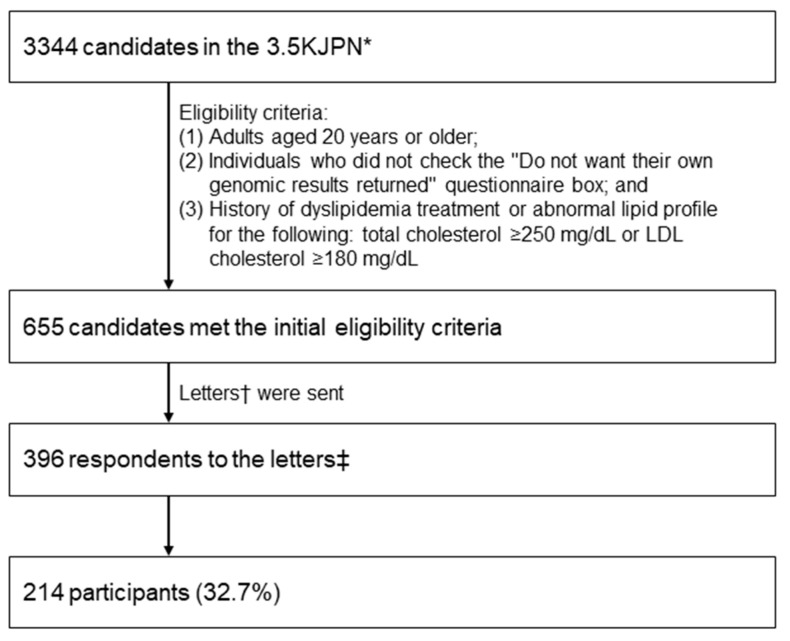
Flow diagram of the study. * Japanese whole-genome reference panel of 3554 samples, including 3344 samples from individuals who participated in the Tohoku Medical Megabank Project led by the Tohoku Medical Megabank Organization at Tohoku University (Miyagi Prefecture, Japan) and the Iwate Tohoku Medical Megabank Organization at Iwate Medical University (Iwate Prefecture, Japan). † Request to participate in this pilot study to receive genomic results targeting monogenic FH (*LDLR*, *PCSK9*, and *APOB*). ‡ Individuals who did not intend to participate but later decided to participate were excluded from this study.

This study was conducted as an adjunct study of the TMM Project [7]. Participants were selected from the 3554 Japanese genome reference panel (3.5KJPN) established within the TMM Project [8]. We accessed their genomic data and returned the results of individual genomic results for monogenic FH based on their requests to proceed to the next step [9]. The eligibility criteria were as follows: (1) ≥20 years of age, (2) unchecked “Do not wish genomic results returned” questionnaire box, and (3) a history of dyslipidemia treatment, or total cholesterol ≥ 250 mg/dL or LDL-C ≥ 180 mg/dL. We invited 655 eligible candidates and 223 (33.5%) expressed their interest to join this pilot study, in which genomic test results for monogenic FH (*LDLR*, *PCSK9*, and *APOB*) would be returned. Furthermore, 215 participants attended the genetics workshop before providing informed consent, and all agreed to participate in the FH pilot study (Figure 1). One participant who did not intend to participate in this study (but later wished to participate) was excluded, and the final number of participants was 214 (32.7%).

The genetics workshop was held at eight assessment centers located in Miyagi prefecture and Iwate prefecture between December 2016 and February 2017 and between September 2017 and November 2017, and was conducted according to a previous study [9], with some modifications [10]. Briefly, the workshop addressed knowledge related to basic genetics and FH, including types (heterozygous or homozygous), incidence, inheritance pattern, natural history, treatments, and therapy. The workshop lasted for 30 min and included PowerPoint slides [10]. After the workshop, at least a three-generation pedigree including the first-, second-, and third-degree relatives of participants and information on their maternal and paternal relatives (and affected and unaffected relatives, especially those with dyslipidemia, heart disease, and stroke) were collected by medical geneticists and/or certified genetic counselors during face-to-face genetic counseling sessions from participants who allowed us to disclose their family history.

### 2.2. Questionnaire

The questionnaires comprised 22 preference/intention-based questions (agree/somewhat agree/neither agree nor disagree/somewhat disagree/disagree). To assess the preferences/intentions of the participants regarding the FH genetic test results, we asked the participants with whom they intended to share their results with and for which family members these results would be considered helpful in managing their health. Participants answered the questionnaires at the venue after the workshop. The genetic test results were not available to the participants at the time of answering the questionnaire after the workshop.

### 2.3. Statistical Analysis

The family health histories and pedigrees were assembled and analyzed using the f-tree software (version 4.0.2; http://iwate-megabank.org/en/genetic/, accessed on 15 February 2024), as described previously [11]. The survey data were statistically analyzed using the R software version 3.5.1, with *p* < 0.05 being considered to reflect a statistically significant difference. Demographic data were summarized using descriptive statistics. We conducted a logistic regression analysis with a forward–backward stepwise selection method to calculate odds ratios (ORs) and 95% confidence intervals (CIs) for outcomes of preferences/intentions regarding FH genetic test results associated with gender, age, one’s own medical history (heart disease), and the medial histories of first-degree relatives (dyslipidemia, heart disease, and stroke).

### 2.4. Ethical Considerations

This study was conducted in accordance with the Declaration of Helsinki, and the protocol was approved by the ethics committees of the participating institutions (approval Nos. 2016-20 [Tohoku University, Japan] and HGH28-16 [Iwate Medical University, Japan]). Written informed consent was obtained from all participants.

## 3. Results

### 3.1. Participant Characteristics

Participant characteristics are shown in Table 1. The male-gender rate and median age of the participants were 33.2% and 67 years, respectively. All 214 final participants (195 from Miyagi and 19 from Iwate) answered the questionnaire and wished for their FH genetic results to be returned. All participants had dyslipidemia, whereas 21 (9.8%) presented with different heart diseases, and 3 (1.4%) had experienced a stroke.

### 3.2. Preferences and Intentions Regarding the FH Genetic Test Results

The preferences and intentions of the participants are shown in Table 2. The majority of participants indicated that they wanted to share their genetic test result, regardless of whether they were positive or negative, with their partner, children, grandchildren, and siblings (51.4–84.1%). Furthermore, they indicated that the result would be useful for managing their own health as well as that of their children, grandchildren, and siblings (66.4–98.1%).

### 3.3. Family Health History

Pedigrees and family health histories were disclosed by 186 participants (86.9%). In total, 2212 relatives were identified, including 1,196 first-degree relatives, 967 second-degree relatives, and 49 third-degree relatives (Table S1). The median number of first-degree relatives disclosed was 6, with a range of 2–12. The familial medical histories showed that 167 (7.5%) relatives had dyslipidemia, 110 (5.0%) had a different heart disease, and 103 (4.7%) had strokes (Table S1). Ninety-five participants (44.4%) had first-degree relatives with dyslipidemia. None of the participants with no first-degree relatives with dyslipidemia reported second- or third-degree relatives with dyslipidemia. Sixty-six participants (30.8%) had first-degree relatives with heart disease, and fifty-eight (27.1%) had first-degree relatives with strokes (Table 1).

### 3.4. Associations between Participant Characteristics and Preferences/Intentions Regarding the FH Genetic Test Results

Table 3 shows the OR results for preferences/intentions regarding the FH genetic test results by participant characteristics. The logistic regression analysis with a forward–backward stepwise selection method revealed that a dyslipidemia history of first-degree relatives significantly influenced preferences and intentions regarding the FH genetic test results. Specifically, this history impacted the desire to share positive genetic test results with siblings (OR = 2.22; 95% CI: 1.20–4.15; *p* < 0.05), the perception that positive results would be useful for managing their sibling’s health (OR = 1.98; 95% CI: 1.05–3.79; *p* < 0.05), the intention to share negative results with siblings (OR = 2.45; 95% CI: 1.35–4.51; *p* < 0.01), and the belief that negative results would be useful for managing their sibling’s health (OR = 2.01; 95% CI: 1.09–3.76; *p* < 0.05). A heart-disease history of first-degree relatives significantly influenced the desire to share negative genetic test results with grandchildren (OR = 1.93; 95% CI: 1.05–3.62; *p* < 0.05), whereas a stroke history of first-degree relatives had a significant negative impact on the desire to share the result with other family members (OR = 0.46; 95% CI: 0.23–0.90; *p* < 0.05). The male gender had a significant negative impact on the perception that negative results would be useful for managing their children’s health (OR = 0.38; 95% CI: 0.16–0.90; *p* < 0.05). Being aged 65 years or older had a significant negative impact on the perception that positive or negative results would be useful for managing their parent’s health (OR = 0.36; 95% CI: 0.19–0.66; *p* < 0.01; for positive results) (OR = 0.41; 95% CI: 0.22–0.74; *p* < 0.01; for negative results). We did not include the characteristic of “Health history of first-degree relatives’ stroke” as an explanatory variable in the logistic regression analysis because its frequency showed a significant imbalance (only 3 of 183 participants had a family history of stroke).

## 4. Discussion

We surveyed 214 participants who had a history of dyslipidemia treatment or abnormal lipid profiles and wished for their FH genetic results to be returned. More than half of the participants wanted to share the results with their partner, children, grandchildren, and siblings, regardless of whether the results were positive or negative, and thought that the result would be useful for managing their own health and that of their children, grandchildren, and siblings.

Specifically, more than 80% of the participants agreed or somewhat agreed to share the results with their partner and children, which indicates a high level of openness in communicating their results within the immediate family. Moreover, more than half of the participants were inclined to share the results with their grandchildren, reflecting a broader concern for the well-being of extended family members. This recognition of the usefulness of genetic test results underscores the growing awareness of genetic information in health management across different generations.

The willingness to share test results and the perceived usefulness thereof reflect an understanding of the relevance of a comprehensive approach towards familial health, further emphasizing the importance of genetic information in preventive healthcare and personal health-management strategies. These findings highlight a prevalent attitude towards genetic testing, where sharing information is seen as a personal choice and a collective family matter, indicative of a proactive approach to familial health and well-being. The data also suggest that genetic test results are considered valuable not only for the participants themselves but also by serving as a tool for managing and planning the health of their family members, spanning multiple generations.

Our findings illuminate the dynamics between family health history, specifically dyslipidemia in first-degree relatives, and the preferences and intentions regarding FH genetic test results. The significant correlation between a family history of dyslipidemia and the willingness to share positive and negative FH genetic test results, especially with siblings, underscores the role of the familial context in shaping attitudes toward genetic-information sharing and health management.

The strong association between the health history of first-degree relatives with dyslipidemia and the preference to share FH genetic test results highlights the perceived importance of gene information in familial health contexts. This finding aligns with a previous study that emphasized the influence of family health history on health-related decisions and perceptions [12]. The increased OR for sharing positive and negative results with siblings suggests the recognition of the shared genetic risks among immediate family members.

The tendency of male participants and those older than 65 to be less inclined to see the value of negative genetic test results in managing the health of children and parents aligns with findings from previous studies [13,14]. We noted that attitudes towards genetic testing vary with age and gender, with younger people showing more interest and positive attitudes [13,15,16]. Another study comparing attitudes toward genetic testing among various demographic groups revealed variations in awareness and attitudes based on gender and other factors [14]. These studies collectively highlight the significant influence of demographic factors such as age and gender on genetic-testing perceptions, emphasizing the need for tailored communication strategies in genetic counseling.

Over the past three decades, advances in human genetics have dramatically improved health outcomes. Novel genetic-testing methods have been pivotal for diagnosis, prognosis, therapy, safety, screening, and risk assessment, further highlighting the importance of understanding and applying genetic information for personalized healthcare [17,18].

Genome-wide association studies have uncovered thousands of genetic variants associated with various traits and diseases. This has revealed widespread pleiotropy, where a single genetic variant can influence multiple traits. Such discoveries are vital for health management and could extend the healthy lifespan [19]. Particularly, a study focusing on coronary events reported a higher relative risk in those with high genetic predispositions than in those with a low risk. This emphasizes the necessity for an awareness of genetic risk factors in managing specific conditions such as coronary disease and prompting a shift towards more proactive health management [5].

Integrating genomics into healthcare leads to a proactive, preventive health model with personalized treatment strategies [20,21,22]. This transition is anchored in the understanding of genetic risks, which is essential for devising effective health-management plans [23]. Moreover, understanding genetic risk can empower individuals to make informed health decisions, akin to practicing defensive driving [24]. This analogy underscores the proactive aspect of health management, especially when informed by genetic predispositions.

Collectively, these insights and studies advocate for proactive health management at both individual and family levels. Sharing genetic test results, particularly for conditions such as FH, has significant implications for preventive health strategies. This not only influences personal health decisions but also extends to family-wide lifestyle choices and preventive measures, emphasizing the role of genetic understanding in shaping health across generations. Our findings lend support to the adoption of a family-centered approach in genetic counseling [25]. Recognizing the shared genetic risks within families, it becomes imperative that genetic counseling extends beyond the individual and encompasses the family unit. In light of the current study’s results, such approaches appear particularly relevant for conditions such as FH, where family history plays a significant role in individual health decisions and attitudes toward genetic testing.

In discussing the influence of familial health history on attitudes toward genetic testing and information sharing, our findings highlight the critical need for integrating genetic testing into familial and personalized healthcare strategies. This need is exemplified by the Estonian population-based biobank study on FH [26]. This study, which involved recalling individuals with FH-related genetic variations for family screenings, emphasizes the vital role of genetic counseling and the necessity of early intervention in managing FH effectively. Such approaches bolster the argument presented in our study for adopting family-centered healthcare strategies and the potential advantages of disseminating genetic information within families, which will ultimately enhance health-management outcomes.

While this study offers valuable insights into how family health history influences attitudes toward genetic testing and information sharing, it has several significant limitations. One of the main limitations is the sample size and scope. There were twice as many female participants as male participants. The findings are derived from 214 participants, mainly from Japan’s Miyagi and Iwate prefectures. This regional focus, the relatively small number of participants, and the disparity in the distribution of male and female participants may affect the generalizability of our results. To expand the applicability of the findings, future studies should include a more extensive and diverse participant pool from various geographic locations and cultural backgrounds. Another fundamental limitation is the data-collection method, which primarily involved questionnaires and face-to-face genetic-counseling sessions. While these methods are effective, they are susceptible to self-reporting biases or selective recall. Incorporating additional data collection methods, such as longitudinal tracking or integrating medical record data, could offer a more comprehensive and nuanced understanding of participants’ attitudes and behaviors. Furthermore, the study did not explore in depth the psychological mechanisms underlying the preferences and intentions of the participants. Investigating these psychological aspects, including risk perception, health literacy, and emotional responses to genetic information is crucial for a deeper understanding of the factors driving these decisions [27,28]. Despite participants expressing a strong intention to share their genetic test results with family members and to use them for health management, it is critical to acknowledge that our study did not verify whether these intentions translated into actual behaviors, such as sharing results and initiating cascade testing within families. Questions regarding the psychological mechanisms driving these intentions highlight the need for further investigation into the actual implementation of these practices. Consequently, future research is needed to track the follow-up actions of individuals who are willing to share genetic-testing information and evaluate the effectiveness of genetic counseling in facilitating these actions. Such research will illuminate how psychological intentions are transformed into tangible health-management actions in familial settings. It is yet to be determined whether the results obtained for a monogenic disease with obvious clinical manifestations (e.g., hyperlipidemia from birth in the case of FH) will generalize to cases of monogenic diseases without apparent clinical manifestations (e.g., hereditary breast and ovarian cancer syndrome before carcinogenesis) or multifactorial diseases, which involve more complex risk factors. Future studies should consider symptom visibility, disease severity, and perceived risk levels. An area not investigated in this study is the impact of living arrangements with family members on sharing and using genetic information. Understanding how physical proximity and sharing daily living area as well as having daily interactions with family members influence the willingness to share and the applicability of the genetic test results could provide critical insights for personalized health-management strategies.

By addressing these limitations in future research, we can not only enhance the findings of this study but also broaden the knowledge base in the field of genetic counseling and family health management. The ultimate goal within the TMM Project is to effectively utilize genomic results for individual health management and comprehensive family health planning, adapting our strategies to the evolving landscape of genetic medicine.

In summary, surveying 214 participants, our study demonstrated a notable willingness to share positive and negative FH genetic test results, particularly with siblings. More than 80% of participants planned to share positive results with immediate family, and 98.1% acknowledged the utility of these results for personal health-management. These findings highlight the significance of family health history in decision making regarding genetic testing and health-management strategies. Broader, more diverse research is required to validate these findings.

## Figures and Tables

**Table 1:** Participant characteristics (*n* = 214).

		*n*	Proportion
Gender	Male	71	33.2%
	Female	143	66.8%
Age	Median (mean, range), years	67	(64.4, 35–90)
	≥65 years	124	57.9%
	<65 years	90	42.1%
Wished for their familial hypercholesterolemia genetic results to be returned	Yes	214	100.0%
	No	0	0.0%
Medical history of the participants	Dyslipidemia	214	100.0%
	Heart disease	21	9.8%
	Stroke	3	1.4%
Disclosed their pedigrees and family health histories	Yes	186	86.9%
	No	28	13.1%
Number of disclosed first-degree relatives	Median (range)	6	(2–12)
Participants with first-degree relatives with the indicated disease	Dyslipidemia	95	44.4%
	Heart disease	66	30.8%
	Stroke	58	27.1%

**Table 2:** Preferences and intentions of participants (*n* = 214).

Preferences and Intentions			Number of Answers (%)			
			Agree and Somewhat Agree		Disagree, Somewhat Disagree, Neither Agree nor Disagree, and Missing Information	
If the genetic test result was *positive* (pathogenic variant detected):	I want to share the result with:	my partner	180	84.1%	34	15.9%
		my children	175	81.8%	39	18.2%
		my grandchildren	110	51.4%	104	48.6%
		my siblings	140	65.4%	74	34.6%
		my other family members	80	37.4%	134	62.6%
	The result will be useful for managing the health of:	my own health	210	98.1%	4	1.9%
		my children’s health	192	89.7%	22	10.3%
		my grandchildren’s health	151	70.6%	63	29.4%
		my parent’s health	109	50.9%	105	49.1%
		my sibling’s health	150	70.1%	64	29.9%
		the health of my other family members	97	45.3%	117	54.7%
If the genetic test result was *negative* (no pathogenic variant detected):	I want to share the result with:	my partner	177	82.7%	37	17.3%
		my children	175	81.8%	39	18.2%
		my grandchildren	119	55.6%	95	44.4%
		my siblings	136	63.6%	78	36.4%
		my other family members	85	39.7%	129	60.3%
	The result will be useful for managing the health of:	my own health	205	95.8%	9	4.2%
		my children’s health	188	87.9%	26	12.1%
		my grandchildren’s health	142	66.4%	72	33.6%
		my parent’s health	106	49.5%	108	50.5%
		my sibling’s health	143	66.8%	71	33.2%
		the health of my other family members	96	44.9%	118	55.1%

**Table 3:** Odds ratios for preferences/intentions regarding the FH genetic test results based on participant characteristics (*n* = 186).

Characteristics		Outcomes ^a^								
		If the Genetic Test Result Was *Positive* (Pathogenic Variant Detected):				If the Genetic Test Result Was *Negative* (No Pathogenic Variant Detected):				
		I Want to Share the Result with:		The Result Will Be Useful for Managing the Health of:		I Want to Share the Result with:		The Result Will Be Useful for Managing the Health of:		
		My Siblings	My Other Family Members	My Parent’s Health	My Sibling’s Health	My Grandchildren	My Siblings	My Children’s Health	My Parent’s Health	My Sibling’s Health
Gender ^b^	*B* (S.E.)							–0.97 (0.44)		
	Wald							4.91		
	*p* value							0.0268 *		
	OR							0.38		
	95% CI							0.16–0.90		
Age ^c^	*B* (S.E.)			–1.03 (0.31)					–0.90 (0.31)	
	Wald			10.78					8.40	
	*p* value			0.0010 †					0.0038 †	
	OR			0.36					0.41	
	95% CI			0.19–0.66					0.22–0.74	
Medical history of the participants’ heart disease ^d^	*B* (S.E.)									
	Wald									
	*p* value									
	OR									
	95% CI									
Health history of first-degree relatives’ dyslipidemia ^d^	*B* (S.E.)	0.80 (0.31)	0.49 (0.31)	0.68 (0.31)	0.68 (0.33)		0.89 (0.31)		0.68 (0.31)	0.70 (0.32)
	Wald	6.41	2.47	4.84	4.39		8.46		5.00	4.88
	*p* value	0.0113 *	0.1157	0.0277 *	0.0361 *		0.0036 †		0.0253 *	0.0271 *
	OR	2.22	1.63	1.97	1.98		2.45		1.98	2.01
	95% CI	1.20–4.15	0.89–3.01	1.08–3.62	1.05–3.79		1.35–4.51		1.09–3.63	1.09–3.76
Health history of first-degree relatives’ heart disease ^d^	*B* (S.E.)					0.66 (0.31)				
	Wald					4.40				
	*p* value					0.0359 *				
	OR					1.93				
	95% CI					1.05–3.62				
Health history of first-degree relatives’ stroke ^d^	*B* (S.E.)	0.59 (0.35)	−0.77 (0.35)							
	Wald	2.78	4.84							
	*p* value	0.0955	0.0278 *							
	OR	1.80	0.46							
	95% CI	0.92–3.65	0.23–0.90							

Logistic regression analysis with a forward–backward stepwise selection method; S.E.: standard error; OR: odds ratio; CI: confidence interval; FH: familial hypercholesterolemia; ^a^ disagree, somewhat disagree, neither agree nor disagree, and missing information coded 0, agree and somewhat agree coded 1; ^b^ female coded 0, male coded 1; ^c^ younger than 65 years coded 0, 65 years or older coded 1; ^d^ no history coded 0, having a history coded 1; * *p* < 0.05; † *p* < 0.01. All characteristics examined through stepwise selection are presented in the table. Blank cells represent that these characteristics were not selected as explanatory variables for the outcome.

## Data Availability

The datasets used and analyzed in the current study are available from the corresponding author upon reasonable request.

## Supplementary Materials

The following supporting information can be downloaded at: https://www.mdpi.com/article/10.3390/genes15030384/s1, Table S1: Relatives’ characteristics (*n* = 2212).
